# The Association Between Delirium Upon Admission to a Rehabilitation Hospital and Motor Rehabilitation Outcomes Among Hip Fracture Surgery Patients: A Historical Cohort Study

**DOI:** 10.3390/jcm13237394

**Published:** 2024-12-04

**Authors:** Anna Balzer, Anne Marie Novak, Pnina Marom, Oren Schwartz, Michael Brik, Katia Slutzki, Rafi J. Heruti, Rachel Dankner

**Affiliations:** 1 Reuth Tel Aviv Rehabilitation Hospital, Tel Aviv 6772830, Israel; a@22i.info (A.B.); anne.novak@reuth.org.il (A.M.N.); pnina.marom@reuth.org.il (P.M.); oren.schwartz@reuth.org.il (O.S.); michael.brik@reuth.org.il (M.B.); doc1@reuth.org.il (K.S.); heroti@reuth.org.il (R.J.H.); 2 Department of Physical Medicine and Rehabilitation, School of Medicine, Faculty of Medical and Health Sciences, Tel Aviv University, Tel Aviv 6997801, Israel; 3 Department of Epidemiology and Preventive Medicine, School of Public Health, Faculty of Medicine and Health Sciences, Tel Aviv University, Tel Aviv 6997801, Israel

**Keywords:** delirium, hip fracture, rehabilitation, older adults, geriatrics

## Abstract

**Background**: Delirium is a common neuropsychiatric syndrome characterized by the acute and fluctuating impairment of cognition, attention, and consciousness, which is prevalent in older adults following surgical procedures. Despite the recognized impact of delirium on recovery, its specific effects on motor rehabilitation outcomes in the geriatric population remain underexplored. This historical cohort study aimed to evaluate the association between the presence of delirium upon admission to a rehabilitation hospital and the motor functional gain at discharge among older patients following hip fracture surgery. **Methods**: The collected data included socio-demographic characteristics, comorbidities, medications, Mini-Mental State Examination (MMSE) scores, and the Functional Independence Measure (FIM). Motor rehabilitation outcomes were assessed using Motor Absolute Functional Gain (mAFG), the Montebello Rehabilitation Factor Score (mMRFS), and Rehabilitation Efficiency (mRE). **Results**: Of the 143 hip fracture patients admitted for rehabilitation, 38 (26.6%) were diagnosed with delirium. Patients with delirium had lower MMSE scores (18.1 ± 5.8 vs. 22.4 ± 6.0, *p* < 0.001), higher benzodiazepine prescription rates (50.0% vs. 14.3%, *p* < 0.001), and longer lengths of stay in acute care and rehabilitation (42.7 ± 10.4 vs. 37.3 ± 11.2 days, *p* = 0.01). Despite significant improvements in the FIM scores for both groups (*p* < 0.001), patients with delirium had lower mAFG (11.87 ± 7.26 vs. 15.91 ± 8.73, *p* = 0.01), mMRFS (0.22 ± 0.14 vs. 0.31 ± 0.15, *p* = 0.001), and mRE (0.28 ± 0.17 vs. 0.44 ± 0.25, *p* < 0.001). However, the multivariate regression models showed no association between delirium and functional improvement after adjusting for confounders. **Conclusions**: While both patients with and without delirium showed improvement in their motor functions by the time they were discharged from a rehabilitation hospital, patients with delirium showed lower absolute and relative improvements. Tailored programs addressing the special needs of patients with delirium after hip fracture surgery may enhance outcomes for this vulnerable population. A specialized, multidisciplinary approach tailored to the patient’s cognitive status and overall condition is key to maximizing the recovery of older hip fracture patients with delirium.

## 1. Introduction

Delirium is a neuropsychiatric syndrome characterized by the acute and fluctuating impairment of cognition, disorientation, and the development of perceptual disturbance [1]. The patient may have slurred speech, and a reduced ability to focus and maintain attention [1]. The highest incidence rates of delirium are reported in intensive care units and postoperative care settings [2]. Delirium continues to affect 44.7% of patients at discharge from acute care hospital, and 32.8%, 25.6%, and 21% at one, three, and six months afterwards, respectively [3]. The presence of delirium is associated with an increased risk of in-hospital falls and higher mortality [4]. It is also associated with a prolonged length of hospital stay and an increase in the cost of hospitalization [5,6].

Delirium is the most frequent complication after hip fracture surgery, with incidence rates ranging between 16% and 39% [7,8]. Hip fractures are among the major causes of hospitalization in older adults and the second most common fracture for men and women above 65 years of age [9]. In the United States, the annual incidence of hip fractures per 100,000 people is estimated to be 197 to 201 for men and 511 to 553 for women [9]. Worldwide, the total number of hip fractures is expected to be greater than 6 million by the year 2050 [9,10]. In developed countries, almost half of all reported hip fractures occur in patients aged 80 or above [5]. More than half of hip fracture patients do not regain mobility in the first postoperative year and about 20% become immobile [11]. Hip fractures in older adults are associated with a mortality rate of 8.1% at 30 days and 21.6% at one year after fracture [12,13]. According to the Israeli Central Bureau of Statistics, about 6000 Israelis sustain a hip fracture per year, a rate of 72.3 per 100,000; of them, about 80% undergo a surgical procedure [14].

An increasing proportion of patients admitted to general hospitals with hip fractures have an underlying cognitive impairment or have been diagnosed with dementia [15]. While the presence of cognitive impairment was previously an exclusion criterion for rehabilitation [16,17], it has been found that rehabilitation offered in post-acute or community settings is beneficial for older adults with cognitive impairment post-hip fracture [18]. In a 2014 study conducted in a rehabilitation hospital, both patients with and without delirium saw an improvement in their Functional Independence Measure (FIM), which is used to assess and grade the functional status of a person based on the level of assistance he or she requires, although the improvement was slower and smaller among patients with delirium [19]. However, the results did not take into account the adjusted rehabilitation outcome, which reflects the patients’ rehabilitative potential, and the efficiency of the rehabilitation treatment, i.e., the speed and effectiveness of the rehabilitation process; the results were reported without adjusting for confounders. The purpose of this study was to evaluate the association between the presence of delirium upon admission to a rehabilitation–geriatric hospital and the motor functional gain of older patients after hip fracture surgery.

## 2. Materials and Methods

This was a historical cohort study, based on the electronic medical records of patients hospitalized at the Reuth Tel-Aviv Rehabilitation Hospital, Israel. The multidisciplinary rehabilitation treatment offered at the hospital includes individually tailored physical and occupational therapy, speech therapy, nutritional management, as well as ongoing psychological assessments, provided in conjunction with standard medical care. The study protocol was approved by the Hospital’s Institutional Review Board (approval number: RRH-005-19, granted on 16 May 2019). The relevant checklist for Strengthening the Reporting of Observational studies in Epidemiology (STROBE) is provided as a Supplementary Materials.

The cohort included all men and women 65 years of age or older admitted for rehabilitation at our geriatric departments between January 2016 and December 2018 following hip fracture surgery. Delirium, the exposure variable, may have been recorded in the letter of discharge from the acute care hospital, where the hip fracture repair surgery took place, or diagnosed during hospitalization for rehabilitation. 

### 2.1. Delirium Assessment

The presence of delirium was determined based on the diagnostic criteria for delirium according to DSM-V (Supplementary Materials Table S1) or the Confusion Assessment Method (CAM) (Supplementary Materials S2). CAM is a diagnostic instrument used for the identification of delirium. The instrument assesses the presence, severity, and fluctuation of 9 delirium features: acute onset, inattention, disorganized thinking, altered level of consciousness, disorientation, memory impairment, perceptual disturbances, psychomotor agitation or retardation, and an altered sleep–wake cycle [20]. The validated Hebrew version of the questionnaire was used by trained physicians during the patients’ evaluation.

### 2.2. Exclusion Criteria

**Figure 1:**
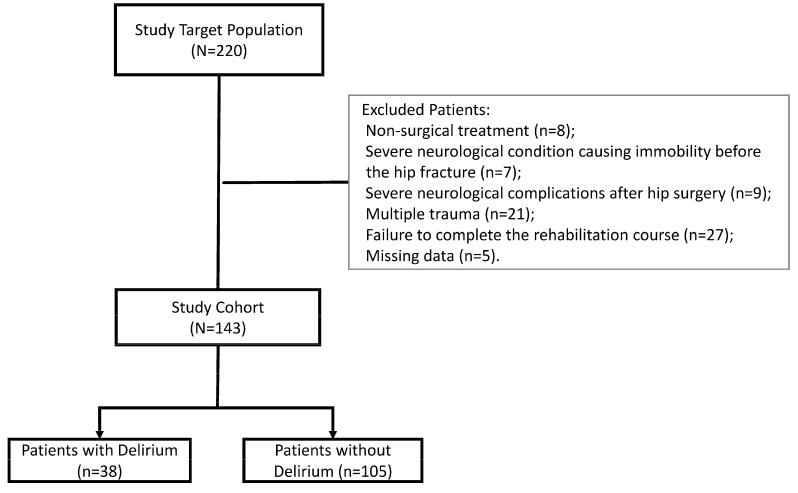
Study cohort flow chart.

The exclusion criteria for this study were as follows: (1) non-surgical treatment for hip fracture; (2) severe neurological condition that resulted in immobility prior to the hip fracture (e.g., stroke, Alzheimer’s, Parkinson’s); (3) severe neurological complications after hip fracture surgery (e.g., stroke); (4) multiple trauma concomitant to the hip fracture; and (5) failure to complete the rehabilitation course, the possible causes of which include re-hospitalization in the acute care hospital or refusal to undergo rehabilitation. Figure 1 depicts the study population.

### 2.3. Rehabilitation Outcomes

Study outcomes were based on the motor Functional Independence Measure (FIM) [21]. The motor FIM, which contributes to the overall FIM score, monitors changes in a patient’s motor functional abilities during rehabilitation. It evaluates the following 13 functional abilities of the patient: Eating; Grooming; Bathing; Dressing—Upper and Lower Body; Toileting; Bladder Management—Level of Assistance; Bowel Management—Level of Assistance; Transfers: Bed, Chair, Wheelchair; Transfers: Toilet; Transfers: Tub or Shower; Locomotion: Walk/Wheelchair; Locomotion: Stairs; Comprehension; and Expression. Each of the abilities is rated on a 7-point ordinal scale. Higher scores indicate greater independence in performing the task associated with each item. The total score for the motor FIM subscale ranges from 13 to 91.

The study utilized a set of 3 selected rehabilitation outcomes, i.e., motor functional improvement and mobility gain, as suggested by Koh et al., [22] based on the motor FIM; these included the following:

(a) A measure assessing the difference between the motor FIM score at admission and the motor FIM score at discharge from the rehabilitation hospitalization: Motor Absolute Functional Gain (mAFG) = (Discharge mFIM − Admission mFIM).
(b) A measure of the relative gain in function during an inpatient rehabilitation stay: the Motor Montebello Rehabilitation Factor Score (mMRFS) = [mAFG/(mMaximum Possible FIM − mAdmission FIM)] × 100. It provides a percentage-based evaluation of functional improvement relative to the maximum potential improvement in motor FIM (score of 91).
(c) A measure of the speed and effectiveness of the rehabilitation process: Motor Rehabilitation Efficiency (mRE) = mAFG/Length of Stay.

Additionally, we collected data about the participants’ sociodemographic characteristics, including their place of residence (home vs. institution), length of hospital stay (LOS), medication treatment, cognitive function (measured using the Mini-Mental State Examination, MMSE), and comorbidities.

### 2.4. Statistical Analysis

Descriptive statistics were used to describe the study cohort, categorical variables were presented as frequencies (*n*) and percentages (%), and continuous variables were presented as means and standard deviation. The patients’ baseline characteristics were compared according to their delirium status using independent *t*-tests for continuous variables and the chi-square test for categorical variables. Differences in the motor rehabilitation outcomes (mFIM, mAFG, mMRFS, mRE) between patients with and without delirium were examined by independent *t*-tests. Multiple linear regression models were used to examine the association between the presence of delirium at admission (as predictor/exposure variable) and the motor rehabilitation outcomes (as dependent variables), while adjusting for age, sex and variables that were found to be statistically significantly different between people with and without delirium in the univariate analysis: number of days between surgery and admission to rehabilitation, days in a rehabilitation hospital, MMSE score, and medication treatment. The significance level was set to 0.05 with a 95% confidence interval. The statistical power reached 78%, with the calculation based on the observed difference in the Motor Montebello Rehabilitation Score, which was 0.09 points, and an alpha of 0.05. The analysis was conducted using Statistical Package for Social Science (SPSS^®^) (IBM Corp. Armonk, NY, USA) version 28.

## 3. Results

### 3.1. Characterization of Participants

Between January 2016 and December 2018, a total of 220 patients were admitted to the Geriatric Rehabilitation Wards at the Reuth Tel-Aviv Rehabilitation Hospital following hip fracture surgery. Of them, 143 (65%) met the study inclusion criteria, 53 of whom were men (37%) and 90 of whom were women (63%). The mean age of the study participants was 85 ± 5.4 (range 69–100). Half of the patients (*n* = 71, 50%) were diagnosed with an extracapsular fracture and 52 (36%) with an intracapsular fracture. More than half of the patients (65%) received treatment with Closed Reduction and Internal Fixation, while the rest underwent total hip replacement or hemiarthroplasty. The prevalence of delirium upon admission reached around one quarter of the cohort (*n* = 38, 27%).

Aside from dementia, which was significantly more common in patients with vs. without delirium (68.4% vs. 27.6%, *p* < 0.001), no differences were observed between the groups in their age distribution (*p* = 0.75), sex (*p* = 0.97), marital status (*p* = 0.82), residence (*p* = 0.28), type of fracture (*p* = 0.44), and comorbidities (*p*-value between 0.13 and 0.84). However, patients with delirium had lower MMSE admission scores (18.1 vs. 22.4, *p* < 0.001), were more likely to have been prescribed benzodiazepines, as well as a combination of benzodiazepines and opioids (*p* < 0.001), spent more days at the acute care hospital prior to their admission to our rehabilitation hospital (12.8 vs. 8.5 days, *p* < 0.01), and had a longer LOS in rehabilitation (42.7 vs. 37.3 days, *p* < 0.01). Table 1 contains a detailed comparison of the baseline characteristics of the patients with and without delirium.

### 3.2. Motor Functional Improvement

While both groups experienced significant improvements in their functional independence scores following rehabilitation (*p* < 0.001), patients with and without delirium differed in terms of all the motor rehabilitation outcomes assessed. The 3.6-point difference between the groups in mFIM at admission (*p* = 0.07; 95% CI ]−0.31, 7.65]) grew to a 7.7 points difference at discharge (*p* < 0.001; 95% CI [3.13, 12.27]). Patients with delirium had smaller Absolute and Relative Functional Gain (*p* = 0.01 and *p* = 0.001, respectively), as well as significantly smaller mFIM efficiency (*p* < 0.001). The detailed values are presented in Table 2. However, the adjusted multivariate linear regression models showed no statistically significant association between the presence of delirium and motor function outcomes, as detailed in Table 3. Significant associations were found between mFIM at discharge, mAFG, and mMRFS and the number of days between surgery and admission to in-patient rehabilitation. Further, LOS was significantly associated with mFIM at discharge, mAFG and mRE. The simultaneous use of both benzodiazepines and opioids was further associated with lower mAFG, mMRFS, and mRE.

## 4. Discussion

The purpose of this historical cohort study was to evaluate the independent association between the presence of delirium upon admission to a rehabilitation hospital and the success of motor rehabilitation among older patients following hip fracture surgery performed at an acute care hospital. Patients with delirium were hospitalized for a longer time at the acute care hospital prior to admission to rehabilitation, a lower cognitive function according to the MMSE test performed during hospitalization at the rehabilitation center, and a longer rehabilitation LOS. They had higher baseline rates of benzodiazepine treatment and scored somewhat lower on the admission motor FIM (*p* = 0.07). However, while both groups of patients saw improvements in their motor FIM by the time of discharge from inpatient rehabilitation, patients with delirium showed a lower improvement in all four functional motor ability measures. Nevertheless, no significant associations between the presence of delirium and motor rehabilitation outcomes were observed in the multivariate linear regression models. It should be noted that in the linear models, delirium was negatively associated with all four motor FIM outcomes, while adjusting for all other variables, and that failure to reach statistical significance may have resulted from the small sample size of the study. While we included all eligible patients in our medical center in the study, a larger cohort with a greater number of delirium patients would be needed to reach the statistical significance of the differences observed between the study groups in the study outcomes.

Our findings align with previous research suggesting that even though patients with delirium have slower and smaller improvements, rehabilitation can still be beneficial [19,23]. Nevertheless, the finding that the presence of delirium did not show a statistically significant association with motor function outcomes in the multivariate regression models may suggest that other factors, such as the length of inpatient rehabilitation and baseline MMSE scores, may play pivotal roles in recovery. Thus, delirium patients may require tailored rehabilitation programs [24], perhaps with greater emphasis placed on their physiotherapy treatment and specific treatments to enhance their motor function abilities while taking into consideration their cognitive state. The rehabilitation plan should allow some scheduling flexibility and take into consideration the adjustments necessary due to the frequently fluctuating nature of delirium and the subsequent fatigue [25].

The association between a longer pre-rehabilitation hospital stay and delirium noted in this study could be indicative of the more severe initial impact of hip fractures in these patients, possibly exacerbated by their cognitive vulnerabilities. Moreover, the higher prescription rates of benzodiazepines and opioids in patients with delirium are of particular concern. While these medications are often used for managing pain and agitation, they may be the cause of delirium, or could paradoxically contribute to worse outcomes in delirium patients by potentially worsening or prolonging delirium and reducing rehabilitation efficiency [26]. Indeed, we observed a much smaller mFIM efficiency in patients with admission delirium than in patients without it. This complex interplay suggests a need for careful medication management and monitoring during the acute and post-acute rehabilitation phases of recovery in older patients.

This study’s findings are consistent with those from other regions, emphasizing the global relevance of delirium management in hip fracture recovery [27,28]. A notable aspect of this study is its focus on a cohort of older adults (mean age 85), a demographic that is often underrepresented in clinical trials but increasingly relevant given global demographic shifts towards an older population. The study’s findings underscore the necessity of tailored rehabilitation protocols that consider cognitive impairment and the potential side effects of commonly used medications. A recent meta-analysis analyzed the effectiveness of early rehabilitation interventions designed to prevent delirium among older patients, and concluded that, while general rehabilitation interventions are effective, there is a lack of tailored programs specifically designed for patients with cognitive impairments [29]. Future research should explore the mechanisms by which rehabilitation can be optimized for delirium patients, perhaps by integrating more delirium-specific interventions such as environmental modifications, frequent reorientation activities, multi-disciplinary approaches, and enhanced staff training into delirium management, along with appropriate medication [30]. Furthermore, investigating the role of preventive measures against delirium pre- and post-surgery could significantly enhance patient outcomes and reduce the rehabilitation burden [31].

### Study Limitations

This study presents several limitations. First, the study cohort consists of patients from a single medical center and its external validity may be limited. However, healthcare coverage in Israel is universal and provided by four Health Maintenance Organizations (HMOs). The Reuth Tel Aviv Rehabilitation Hospital works in cooperation with all four HMOs and receives patients from all over the country and with various socio-demographic backgrounds. Furthermore, the delirium rates in our hospital were similar to those reported by other hospitals in Israel [19]. Another limitation of this study is its small sample size, which underpowered our analyses, reaching a power of 78%. In line with the observational historical nature of the study, it was exposed to misclassification bias due to potential inappropriate data entry or diagnosis. Variations in the process of diagnosing delirium in acute care hospitals prior to admission to rehabilitation may have led to the misclassification of the exposure variable, which might have impacted the results, most probably toward the null. Finally, previous studies of the association between delirium and rehabilitation functional outcomes included additional explanatory factors such as sleep deprivation [32], metabolic encephalopathy [32,33] surgical stress responses [33], perioperative hypoxemia and hypotension [34], infections [8], Parkinson’s disease [8], and patients’ behavioral characteristics (specifically, alcohol and tobacco use) [35], which are known to be related to the development of delirium. Unfortunately, this information was not available in this historical cohort study, making the adjustment for these potential confounders impossible within this historical cohort study, subjecting our analysis to residual confounding.

## 5. Conclusions

In this study, we found no significant association between the presence of delirium and motor functional improvement. However, patients with delirium, despite significant improvements in their motor FIM, saw smaller increases in their rehabilitation indices, compared to patients without delirium, following rehabilitation. Furthermore, patients with delirium stayed longer at acute care hospitals following surgery, before admission to rehabilitation, and they presented with higher benzodiazepine prescription rates compared to patients without delirium, along with worse cognitive function. These results suggest that older patients with delirium after hip fracture surgery should not be prevented from admission to rehabilitation, even if their rehabilitation journeys may be different. A specialized, multidisciplinary approach that is tailored to the patient’s cognitive status and overall condition is key to maximizing the recovery potential in older hip fracture patients with delirium. Further, larger studies, adjusting for additional underlying health conditions and social support factors, which could impact rehabilitation outcomes, are called upon.

## Figures and Tables

**Table 1:** Baseline characteristic of 143 elderly patients hospitalized for rehabilitation following hip fracture according to their delirium status upon admission.

Characteristic	With Delirium	Without Delirium	
	*n (%)* or *mean ± SD*	*n (%)* or *mean ± SD*	*p-value* ^a^
*n* (%)	**38 (26.6)**	**105 (73.4)**	
**Sex**			0.97
Female	24 (63.2)	66 (62.9)	
Male	14 (36.8)	39 (37.1)	
**Age**, years	85.1 ± 5.3	85.4 ± 5.5	0.75
Median age [range]	86.0 (69–97)	85.0 (74–100)	
85>	16 (42.1)	58 (55.2)	0.16
85 and older	22 (57.9)	47 (44.8)	
**Marital status**			0.82
Married	13 (34.2)	38 (36.2)	
Unmarried	25 (65.8)	67 (63.8)	
**Residence**			0.28 ^b^
Home	37 (97.4)	90 (90.9)	
Institution	1 (2.6)	9 (9.1)	
**Type of fracture**			0.44
Extracapsular	20 (52.6)	51 (48.6)	
Intracapsular	15 (39.5)	37 (35.2)	
Other	3 (7.9)	17 (16.2)	
**Comorbidities**			
Dementia	26 (68.4)	29 (27.6)	<0.001
Anemia	19 (50.0)	50 (47.6)	0.8
Depression	12 (31.6)	34 (32.4)	0.92
Diabetes mellitus	8 (21.1)	36 (34.3)	0.13
Sleep Disorder	8 (21.1)	20 (19.0)	0.79
Stroke	7 (18.4)	14 (13.3)	0.44
Visual impairment	6 (15.8)	18 (17.1)	0.85
Renal failure	6 (15.8)	18 (17.1)	0.84
Chronic Heart Failure	3 (7.9)	8 (7.6)	0.95
**Selected Medications**			<0.001
Opioids	1 (2.6)	38 (36.2)	
Benzodiazepines	19 (50.0)	15 (14.3)	
Both	16 (42.1)	12 (11.4)	
None	2 (5.3)	40 (38.1)	
**Mini-Mental State Examination** score at admission	18.1 ± 5.8	22.4 ± 6.0	<0.001
**Days**: surgery to admission to rehabilitation	12.8 ± 6.4	9.5 ± 4.6	<0.01
**Length of Stay** in a rehabilitation hospital	42.7 ± 10.4	37.3 ± 11.2	0.01

**Notes:** ^a^ *p*-value derived from comparison analysis of chi-square for categorical variables and *t*-test for continuous variables between patients with and without delirium; ^b^ Calculated via Fisher exact test due to there being fewer than five observations.

**Table 2:** Rehabilitation outcomes of 143 elderly patients hospitalized for rehabilitation following hip fracture according to their delirium status upon admission.

Rehabilitation Outcomes	With Delirium *n* = 38	Without Delirium *n* = 105	
	mean ± SD	mean ± SD	*p*-value ^a^
Motor FIM ^b^ at admission	37.89 ± 9.53	41.56 ± 10.87	0.07
Motor FIM ^b^ at discharge	49.76 ± 10.42	57.48 ± 10.36	<0.001
Motor Absolute Functional Gain ^c^	11.87 ± 7.26	15.91 ± 8.73	0.01
Motor Montebello Rehabilitation Factor Score ^d^	0.22 ± 0.14	0.31 ± 0.15	0.001
Motor Rehabilitation Efficiency ^e^	0.28 ± 0.17	0.44 ± 0.25	<0.001

**Notes:** ^a^ *p*-value derives from comparison analysis of motor functional measurements by *t*-test between patients with and without delirium; ^b^ motor Functional Independence Measurement (mFIM); ^c^ motor Absolute Functional Gain (mAFG) = mFIM at discharge − mFIM at admission; ^d^ motor Montebello Rehabilitation Factor Score (MRFS) = [mAFG/(Maximum Possible mFIM − Admission mFIM)] × 100; ^e^ motor Rehabilitation Efficiency (mRE) = mAFG/duration of stay in a rehabilitation hospital (days).

**Table 3:** Adjusted linear regression models for the association between delirium upon admission and four motor functional measures, at hospital discharge, of 143 patients after a hip fracture surgery.

Variable (Reference Group)	mFIM at Discharge ^a^			Motor Absolute Functional Gain ^b^			Motor Montebello Rehabilitation Factor Score ^c^			Motor Rehabilitation Efficiency ^d^		
	B (SE)	95% Cl	*p*	B (SE)	95% Cl	*p*	B (SE)	95% Cl	*p*	B (SE)	95% Cl	*p*
Constant	62.84 (14.52)	34.03, 91.66	<0.001	3.66 (13.52)	−23.12, 30.30	0.80	0.11 (0.24)	−0.37, 0.60	0.65	0.48 (0.41)	−0.33, 1.30	0.24
Delirium (without delirium)	−1.80 (2.44)	−6.63, 3.05	0.50	−0.25 (2.32)	−4.73, 4.23	0.91	−0.01 (0.04)	−0.08, 0.07	0.87	−0.01 (0.06)	−0.15, 0.12	0.84
Sex (men)	−1.11 (1.73)	−4.45, 2.22	0.50	0.42 (1.60)	−2.67, 3.51	0.80	0.01 (0.02)	−0.05, 0.05	0.98	0.01 (0.04)	−0.08, 0.10	0.89
Age	−0.18 (0.22)	−0.40, 0.23	0.60	0.18 (0.15)	−0.20, 0.40	0.51	0.01 (0.01)	−0.01, 0.01	0.59	0.01 (0.01)	−0.01, 0.01	0.61
Days: surgery to admission to rehabilitation ^d^	−0.34 (0.14)	−0.63, −0.10	<0.05	−0.25 (0.13)	−0.53, 0.01	0.06	−0.01 (0.02)	−0.01, 0.00	0.05	−0.01 (0.01)	−0.01, 0.02	0.16
LOS in rehabilitation	−0.22 (0.12)	−0.40, −0.16	<0.01	0.20 (0.16)	0.05, 0.35	<0.01	0.01 (0.01)	−0.01, 0.04	0.26	−0.01 (0.01)	−0.01, −0.01	0.03
MMSE score	0.65 (0.20)	0.40, 0.93	<0.001	−0.01 (0.12)	−0.26, 0.24	0.95	0.01 (0.01)	−0.01, 0.02	0.12	0.01 (0.02)	−0.01, 0.02	0.1
Medications												
Benzodiazepines	−1.10 (2.62)	−6.34, 4.11	0.71	−3.93 (2.43)	−8.68, 0.96	0.11	−0.05 (0.04)	−0.14, 0.03	0.20	−0.08 (0.07)	−0.23, 0.06	0.25
Benzodiazepines and Opioids	−3.15 (2.90)	−8.87, 2.55	0.33	−7.87 (2.76)	−13.10, −2.47	<0.01	−0.11 (0.04)	−0.21, −0.01	<0.02	−0.16 (0.08)	−0.32, 0.00	0.05
None	2.15 (2.11)	−2.05, 6.26	0.30	−0.75 (1.92)	−4.60, 3.05	0.70	0.01 (0.03)	−0.05, 0.08	0.62	−0.01 (0.05)	−0.12, 0.10	0.85
Adjusted *R*^2^	0.38			0.11			0.14			0.10		
Model’s *p*-value	<0.001			0.01			<0.01			0.01		

**Notes:** ^a^ mFIM: motor Functional Independence Measurement; ^b^ mAFG: motor Absolute Functional Gain = mFIM at discharge − mFIM at admission; ^c^ mMRFS: motor Montebello Rehabilitation Factor Score (MRFS) = [mAFG/(Maximum Possible mFIM − Admission mFIM)] × 100; ^d^ mRE: Rehabilitation Efficiency (RE) = mAFG/duration of stay in a rehabilitation hospital (days).

## Data Availability

The dataset for the current study is available from the corresponding authors upon reasonable request.

## Supplementary Materials

The following supporting information can be downloaded at: https://www.mdpi.com/article/10.3390/jcm13237394/s1, Table S1: Delirium, ICD9-CM codes; Supplementary Materials S2: Diagnostic criteria for delirium according to DSM-V; Table S2: STROBE Statement.
